# TBA_225_, a fusion toxoid vaccine for protection and broad neutralization of staphylococcal superantigens

**DOI:** 10.1038/s41598-019-39890-z

**Published:** 2019-03-01

**Authors:** Arundhathi Venkatasubramaniam, Rajan P. Adhikari, Thomas Kort, Grant C. Liao, Shawn Conley, Laura Abaandou, Shweta Kailasan, Yoshikuni Onodera, Subramaniam Krishnan, Didier M. Djagbare, Frederick W. Holtsberg, Hatice Karauzum, M. Javad Aman

**Affiliations:** 1 Integrated Biotherapeutics, Inc., Rockville, MD USA; 2 Daiichi Sankyo, Tokyo, Japan

## Abstract

Superantigens (SAgs) play a major role in the pathogenesis of *Staphylococcus aureus* and are associated with several diseases, including food poisoning, bacterial arthritis, and toxic shock syndrome. Monoclonal antibodies to these SAgs, primarily TSST-1, SEB and SEA have been shown to provide protection in animal studies and to reduce clinical severity in bacteremic patients. Here we quantify the pre-existing antibodies against SAgs in many human plasma and IVIG samples and demonstrate that in a major portion of the population these antibody titers are suboptimal and IVIG therapy only incrementally elevates the anti-SAg titers. Our *in vitro* neutralization studies show that a combination of antibodies against SEA, SEB,and TSST-1 can provide broad neutralization of staphylococcal SAgs. We report a single fusion protein (TBA_225_) consisting of the toxoid versions of TSST-1, SEB and SEA and demonstrate its immunogenicity and protective efficacy in a mouse model of toxic shock. Antibodies raised against this fusion vaccine provide broad neutralization of purified SAgs and culture supernatants of multiple clinically relevant *S*. *aureus* strains. Our data strongly supports the use of this fusion protein as a component of an anti-virulence based multivalent toxoid vaccine against *S*. *aureus* disease.

## Introduction

*Staphylococcus aureus (SA)* is a leading cause of hospital and community-associated infections worldwide with no effective vaccines available^1^. The remarkable ability of SA to cause a wide range of diseases from skin and soft tissue infections (SSTI) to life threatening sepsis and pneumonia is in part due to its ability to escape the immune response using a plethora of virulence factors: the superantigenic and pore-forming toxins, coagulase, capsular polysaccharide, adhesins, proteases, and complement inactivating exoproteins^2^. Since its first emergence in the 1960s methicillin-resistant SA (MRSA) has become endemic in healthcare settings, and more recently also within the community, posing a major global challenge^3,4^. There have hence been increasing efforts directed towards the development of vaccines and therapeutics for SA infections. However, to date, no successful vaccine or antibody against SA infections has been developed, and there has been a spate of clinical trial failures on this front^1,5–8^.

Targeting SA toxins represent an alternative approach as “anti-virulence” vaccine for prevention of severe *SA* disease. Staphylococcal pore forming toxins alpha and gamma hemolysins and leukotoxins play critical roles in immune evasion, by killing cells of the first line of defense such as neutrophils, monocytes, and macrophages, providing iron for bacterial growth by lysing red blood cells, or enabling dissemination of bacteria through killing of cells with critical barrier function such as epithelial cells^2^. Pyrogenic superantigens (SAgs) represent a major family of SA toxins composed of staphylococcal enterotoxins (SEs) and toxic shock syndrome toxin 1 (TSST-1). In contrast to conventional antigens that undergo proteolytic processing by antigen presenting cells to be presented as MHC/peptide complex to T cells, SAgs cross-link T cell receptor (TCR) with MHC Class II and activate up to 30% of T cells^9^ leading to a massive release of cytokines and chemokines, enhanced expression and activation of cell-adhesion molecules, increased T-cell proliferation, and eventually T cell apoptosis or anergy. This sequence of events can culminate in toxic shock syndrome (TSS), a life-threatening condition characterized by rash, hypotension, fever, and multisystem dysfunction^10^. Antibodies play an important role in protection against TSS, thus individuals that do not seroconvert towards the offending toxin due to hypo responsive T-cells^11^ and/or T-cell dependent B-cell apoptosis^12^ are more likely to experience recurring bouts. Furthermore, SAgs impact the virulence of SA strains through induction of a local excessive inflammatory response, immune subversion by inducing apoptosis of T and B cells^11,12^, modulating the function of regulatory T cells (Tregs)^13–15^, innate lymphoid cells (ILCs)^16^ and unconventional T cells such as γδ T cells^17,18^, NKT cells^19–21^, and mucosa associated invariant T (MAIT) cells^22^. Besides TSS, SAgs along with other *S*. *aureus* toxins contribute to pneumonia, infective endocarditis, neonatal exanthematous disease, sepsis, and atopic dermatitis^23–25^.

A major challenge for development of vaccines against SAgs is the fact that various SA strains can produce one or more of over twenty superantigens^26^. While the primary sequence identity among SAgs is limited they exhibit considerable structural homology^27^. Consistent with this structural homology some limited antibody cross-reactivity to certain superantigens has been reported^28^. Most people have been exposed to SA and have antibodies to SAgs and other SA toxins^29^. Intravenous immunoglobulin (IVIG) has been used to treat TSS caused by streptococcal and staphylococcal SAgs, however with limited success^30–33^. Thus, developing broadly cross-neutralizing vaccines and immunotherapeutics for SAgs is highly desirable.

Our present study, based on measurement of anti-SAg antibodies in human plasma and IVIG, suggests that patients can benefit from immunization with SAg-based vaccines that elicit an anamnestic antibody response to three superantigens SEA, SEB, and TSST-1. We demonstrate that antibodies against these SAgs, affinity purified from IVIG, exhibit a wide range of cross-neutralizing activity towards various SAgs with the best activity observed when such antibodies were combined. Based on this finding we generated a fusion protein of rationally designed toxoids for SEA, SEB, and TSST-1. Here, we demonstrate that this fusion vaccine (TBA_225_) is free of superantigenic toxicity and elicits highly protective antibody responses that cover a wide range of SAgs produced by various clinically relevant SA strains. Thus, this vaccine is a prime candidate for inclusion into a multivalent *S*. *aureus* vaccine.

## Experimental Procedures

### Bacterial superantigens and endotoxin

Superantigens SEA, SEB, SEC-1, SEC-2, SEC-3, SED, SEE, SEH, SEK, TSST-1, SpeA and SpeC (certified as >95% purity by SDS) were purchased from Toxin Technology (Sarasota, FL), reconstituted with deionized water and stored at −80 °C until use.

### Affinity purification of human anti-SAg antibodies

SEA, SEB and TSST-1 were coupled to agarose beads (1 mg SAg/mL of bead volume) of an Aminolink® plus immobilization column (ThermoScientific, Rockford, IL) following the manufacturer’s protocol. Affinity purification of specific antibodies from commercial IVIG (Omrix Biopharmaceuticals, Nes-Ziona, Israel) was carried out according to manufacturer’s protocol with minor modifications: 50 mL of IVIG was incubated with toxin-coupled beads for 90 minutes at room temperature (RT) with gentle rocking, centrifuged, and the supernatant removed and a fresh 50 mL of IVIG incubated with the beads for another 1 hour and 30 minutes. Elution was performed with glycine HCl pH 2.5 buffer. Eluted fractions were collected in neutralizing buffer, containing 0.1 M Tris at pH 9 (final pH 6–7). 37.5 µl of affinity-purified anti-SEA, -SEB, - TSST-1, in semi-log dilutions (0.02–20 µg/ml) or IVIG in semi log dilutions (2.5–2500 µg/ml) and 37.5 µl of a 1 ng/ml preparation of either SEB, SEC. 1–3, SEE, SEH, SEI, SEK, TSST-1, SpeC, or 2 ng/ml of SED, or 3 ng/ml of SpeA were mixed. To test the synergistic activity of purified polyclonal Abs a combination of anti-SEA, -SEB, and –TSST-1 were used in a semi log dilution ranging from 0.02 to 20 µg/ml along with the same amount of toxin as above.

### Production of fusion constructs

The genes encoding the toxoids were codon optimized, synthesized, cloned into the pET24a (+) expression vector and transformed into BL21(DE3) *E*. *coli* cells. Overnight cultures were expanded in Luria Broth containing kanamycin until a mid-log phase culture (~0.5 OD at 600 nm), at which point the cells were chilled to ~25 °C and induced with 0.3 mM IPTG, followed by overnight culture at 25 °C. The cells were then harvested, weighed, and resuspended in cell lysis buffer (20 mM Tris pH 8.0, 50 mM NaCl, 1 mM EDTA, 0.1% Triton X-100). Lysozyme was added (1 mg/mL), the cells incubated at 37 °C for 30 minutes, and then the partially lysed cells were sonicated. The cell lysate was adjusted to 0.5 M NaCl, and the nucleic acid was precipitated by the addition of polyethyleneimine (PEI) under constant mixing. The PEI pellet was removed by centrifugation, and the supernatant containing the toxoid was subjected to ammonium sulfate (ASO_4_) precipitation. The ASO_4_ pellets were recovered by centrifugation and stored at −80 °C. The ASO_4_ pellets were resuspended and desalted into the capture column equilibration buffer, clarified, and subjected to chromatography over a Poros 50 HS cation exchange column. The column was equilibrated, loaded, washed and eluted using a 40-column volume (CV) gradient from 25 to 1,000 mM NaCl in phosphate buffer at pH 6.5. The column fractions were analyzed by SDS to determine the toxoid containing fractions, the pooled material dialyzed into the next column equilibration buffer and subjected to chromatography over a BioRad ceramic hydroxyapatite (HTP) (40-micron bead) Type I column. The column was equilibrated, loaded, washed and eluted using a 40 CV gradient of 50–1,000 mM NaCl in a phosphate buffer at pH 6.8. The fractions were analyzed by SDS and the pooled HTP fractions were dialyzed into the appropriate storage buffer, filter sterilized, aliquoted and frozen at −80 °C.

The fusion constructs were then characterized by SDS, WB and HPLC. For WB, primary antibody for the sample (rabbit anti-SEA, SEB, TSST pAb) (0.25 µg/ml) and goat anti-rabbit IgG alkaline phosphatase conjugate (Bio-Rad) secondary antibody (1:3000, v/v) were used. For SEC-HPLC, 10–80 µg of TBA_225_ were injected in an Agilent Technologies 1260 Infinity Series instrument using an AdvanceBio SEC. 300 Å 7.8 × 300 mm LC column with a mobile phase of 50 mM sodium phosphate buffer + 150 mM NaCl, pH 7.0 running at a flow rate of 0.5 mL/min. The chromatogram generated by the Agilent OpenLabs software plots absorbance at 280 nm as a function of retention time. All analysis of the peaks was performed by the auto-integrate function in the OpenLabs software.

### Thermal stability

The thermal stability of TBA_225_ and the individual toxoids was determined by differential scanning fluorimetry (DSF) as described previously^34^. Briefly, 5 μL of 10X SYPRO-Orange dye (Invitrogen, Carlsbad, CA, USA) was added to each sample for a final reaction volume of 25 μL. The thermal assay was conducted in a BioRad CFX Connect thermocycler with the temperature ramped from 30 to 99 °C at intervals of 0.1 °C/6 s. The melting temperature (Tm) for each sample is defined as the vertex of the first derivative (dF/dT) of relative fluorescence unit (RFU) values. Bovine serum albumin (Pierce) with a Tm of 60 °C ± 0.0 was used as a control.

### Human cells and plasma samples

Commercially-sourced human peripheral blood mononuclear cells (PBMCs) were collected and isolated, using the Advarra Institutional Review Board (https://www.advarra.com/) with a peer-approved protocol, from heparinized blood of non-study de-identified healthy human donors by Ficoll gradient centrifugation as described elsewhere^35^ and stored in liquid nitrogen until further use. Deidentified plasma samples were received from Omrix Biopharmaceuticals (Nes-Ziona, Israel). All studies involving human samples were performed in accordance with the applicable guidelines and regulations.

### PBMC stimulation profile of toxoids

75 µl of PBMC cell suspension (at 1.5 × 10^5^ cells) with a viability of >95% was then added to a 96-well plate containing the toxoid or toxin to be tested diluted semi-log serially starting at 1000 ng/ml. After incubation at 37 °C in 5% CO_2_-95% air for 48 hours, the plates were centrifuged at 1500 rpm for 10 minutes, culture supernatants harvested and IFNγ concentration (pg/ml) was determined by ELISA (Human IFN-gamma DuoSet, R&D Systems, Minneapolis, Minn.) following the manufacturers’ protocol.

### Serology ELISAs

Serology ELISAs were performed as described previously^36^. Briefly, 96-well plates were coated with 100 ng/well of wild type (WT) proteins overnight at 4 °C. After washing, plates were then blocked for one hour at room temperature (RT) followed by three washes. Plates were incubated for one hour at RT with the test serum samples (diluted semi-log) and washed three times before applying goat anti-mouse IgG (H&L)-HRP (Horse Radish Peroxidase) in starting block buffer. Plates were incubated for one hour at RT, washed, and incubated with TMB (3,3′,5,5′-tetramethylbenzidine) to detect HRP activity for 30 min. Optical density at 650 nm was measured using a Versamax™ plate reader (Molecular Devices, CA). Data analysis for full dilution curves was performed using the SoftmaxPro software version 5.4.5 (Molecular Devices, CA).

### Quantitative ELISA for anti-SAg antibodies in plasma and IVIG

Standard anti-SAg IgGs were established using affinity purified human antibodies against SEA, SEB, SED, TSST-1, SpeA, and SpeC. The purified antibodies were quantified by BCA assay. Standard curves were established using a full dilution of each antibody on wells of a 96 well plate coated with the respective toxins and bound antibodies detected with HRP-conjugated anti-human IgG. The standard curve (4PL) was repeated 10 times with a CV of <20% with respect to the inflection point and lower and upper asymptotes. To measure the concentration of each anti-SAg antibody content in plasma samples they were run in duplicates and at two dilutions along with the respective standard and the concentration of the anti-SAg in the unknown plasma determined using a 4PL curve fit.

### Adsorption studies

SAg cocktail, individual components SEA, STEBVax, TSST-1, TBA and TBA_225_ were incubated with Alhydrogel at various antigen: adjuvant ratios for half hour at RT. After incubation, the antigen-adjuvant mixture was centrifuged, and the supernatant was detected on an SDS gel. Adsorption of antigens to Alhydrogel was indicated by a thin or negligible band of protein visible on the supernatant SDS as compared to control without any Alhydrogel depicted by the non-adsorbed complete antigen band.

### Animal studies

Six to eight-week-old female Balb/c mice were purchased from Charles River (Wilmington, MA). All mouse work was conducted in accordance with protocols approved by Integrated BioTherapeutics’ institutional animal care and use committee (IACUC) and applicable guidelines and regulations.

For prophylactic protection studies in BALB/c mice, 1.25 μg of superantigen was pre-incubated with 125 μg of total IVIG or purified human polyclonal Abs for 1 h at room temperature before intraperitoneal (IP) administration in a total volume of 200 μl. Four hours post injection, SAg toxicity was potentiated with 40 μg of LPS administered intraperitoneally. To evaluate the therapeutic activity of human polyclonal anti-SEB antibodies mice were challenged with 1.25 μg of SEB at t = 0 h and received 40 μg of LPS at t = 4 h via IP route. At t = 6 h, mice were treated with 125 μg of purified human polyclonal anti-SEB antibodies. Mice were monitored for morbidity (weight loss, hunched posture, lethargy, ruffled fur) and mortality over a period of 4 days.

### Generation of rabbit polyclonal sera to TBA_225_

Anti-TBA_225_ polyclonal was generated (GenScript, Piscataway, NJ 08854, USA) using TBA_225_ protein (>95% purity) as an immunogen. Immunizations were done for four rabbits on day 0, 14, 21 with 0.2 mg protein per rabbit with Freud’s Incomplete Adjuvant injected subcutaneously. Test bleeds and production bleeds were performed on day 21 and day 42 (GenScript). Each serum from production bleed was individually characterized for ELISA titer and TNA titers before pooling together. Pooled anti-serum was purified by Protein A affinity chromatography into total IgG and labeled as anti-TBA_225_ polyclonal antibody.

### Superantigen neutralization assay

PBMCs were prepared in the same way as described above. 75 µl of this cell suspension (1.5 × 10^5^ cells) with a viability of >95% was then added to a 96-well plate containing 75 µl of antibody/toxin mixed at 1:1 as follows: semi-log dilutions of sera starting at 1:40 or antibody starting at 1000 µg/ml and a 0.1 ng/ml preparation of SEB, 1 ng/ml of either SEA, TSST-1 or SEC-1, 0.3 ng/ml of SpeC, or 3 ng/ml of SED, SEE, SEK or SEH. Wells containing medium with toxin only served as positive controls. The plates were incubated at 37 °C in an atmosphere of 5% CO_2_-95% air for 48 hours. Plates were centrifuged at 1500 rpm for 10 minutes, culture supernatants harvested and IFNγ concentration (pg/ml) was determined by ELISA. Plates were read at 450 nm using the Versamax plate reader and data was analyzed in Excel.

### Preparation of bacterial supernatants and neutralization using TBA_225_ polyclonal

Overnight grown bacterial culture supernatants in tryptic soy broth (TSB) were normalized based on culture OD at 600 nm and sterile filtered through 0.2 µm filter. Bacterial supernatants were then diluted semi-log fold in the interferon-gamma production assay previously described. Dilutions of the supernatants at which ~1000–3000 pg/ml interferon-gamma response was produced were selected and then tested for neutralization by TBA_225_ rabbit polyclonal antibody as well as by LukF_mut1_ rabbit polyclonal antibody (negative control).

## Results

### Anti-SAg antibodies in human plasma and IVIG

**Figure 1:**
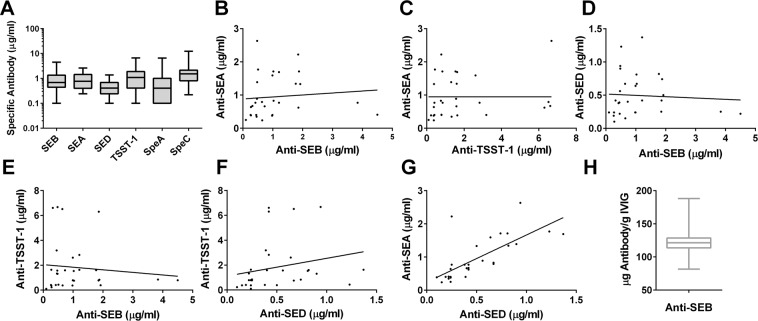
Presence of anti-superantigen antibodies in human plasma and commercial IVIG. (**A**) Range of antibodies against staphylococcal (SEB, SEA, SED TSST-1) and streptococcal (SpeA and SpeC) superantigens in human plasma (n = 30). (**B**–**G**) Correlation of antibody titers to staphylococcal superantigens in human plasma (**H**) Median concentration of anti-SEB polyclonal antibodies in commercially available human IVIG (n = 28).

Given the ubiquity of *S*. *aureus* strains most humans likely have a memory response to staphylococcal antigens such as SAgs due to environmental exposure. A SAg vaccine will likely boost this memory response in an anamnestic fashion. While several groups have reported anti-SAg neutralizing antibodies in IVIG and human serum^37–41^, little is known about the exact quantity of anti-SAg antibodies and their cross-neutralizing profile. Using a quantitative ELISAs to measure human antibodies against four staphylococcal SAgs SEA, SEB, TSST-1, and SED as well as two streptococcal SAgs: streptococcal pyogenic exotoxins A (SpeA) and C (SpeC), we quantified the content of antibodies against these toxins in 30 plasma samples from healthy individuals. As shown in Fig. 1A, the range of plasma antibodies against these toxins was comparable and in the range of 0.1–4.5 (Median 0.7) μg/ml for SEB, 0.24–2.6 (Median 0.77) μg/ml for SEA, 0.1–1.4 (Median 0.4) μg/ml for SED, 0.1–6.7 (Median 1.1) μg/ml for TSST-1, 0.1–6.6 (Median 0.41) μg/ml for SpeA, and 0.22–12.4 (Median 1.5) μg/ml for SpeC. The antibody concentrations against various SAgs generally did not strongly correlate with each other (Fig. 1B–F) suggesting independent exposures to strains expressing various SAgs. An exception was a strong correlation between anti-SEA and anti-SED IgG concentrations (Fig. 1G) suggesting co-exposure or strong cross reactivity. These data suggest that, while there is a wide range of anti-SAg response in individuals as previously reported^37–41^, the level of cross reactivity of the naturally elicited antibodies is fairly limited and may require vaccination.

Human IVIG is used as adjunctive therapy to treat toxic shock induced by staphylococcal and streptococcal superantigens (SAgs) with limited success^30–33^. This may relate to insufficient level of anti-toxin antibodies in IVIG. We evaluated the concentration of anti-SEB IgG in 28 lots of commercial IVIG. The anti-SEB concentrations ranged from 4.1.1 to 9.4 μg/ml (Median 6.1 μg/ml) corresponding to 82–188 μg per gram IVIG (Median 121 μg/g) (Fig. 1H).

**Figure 2:**
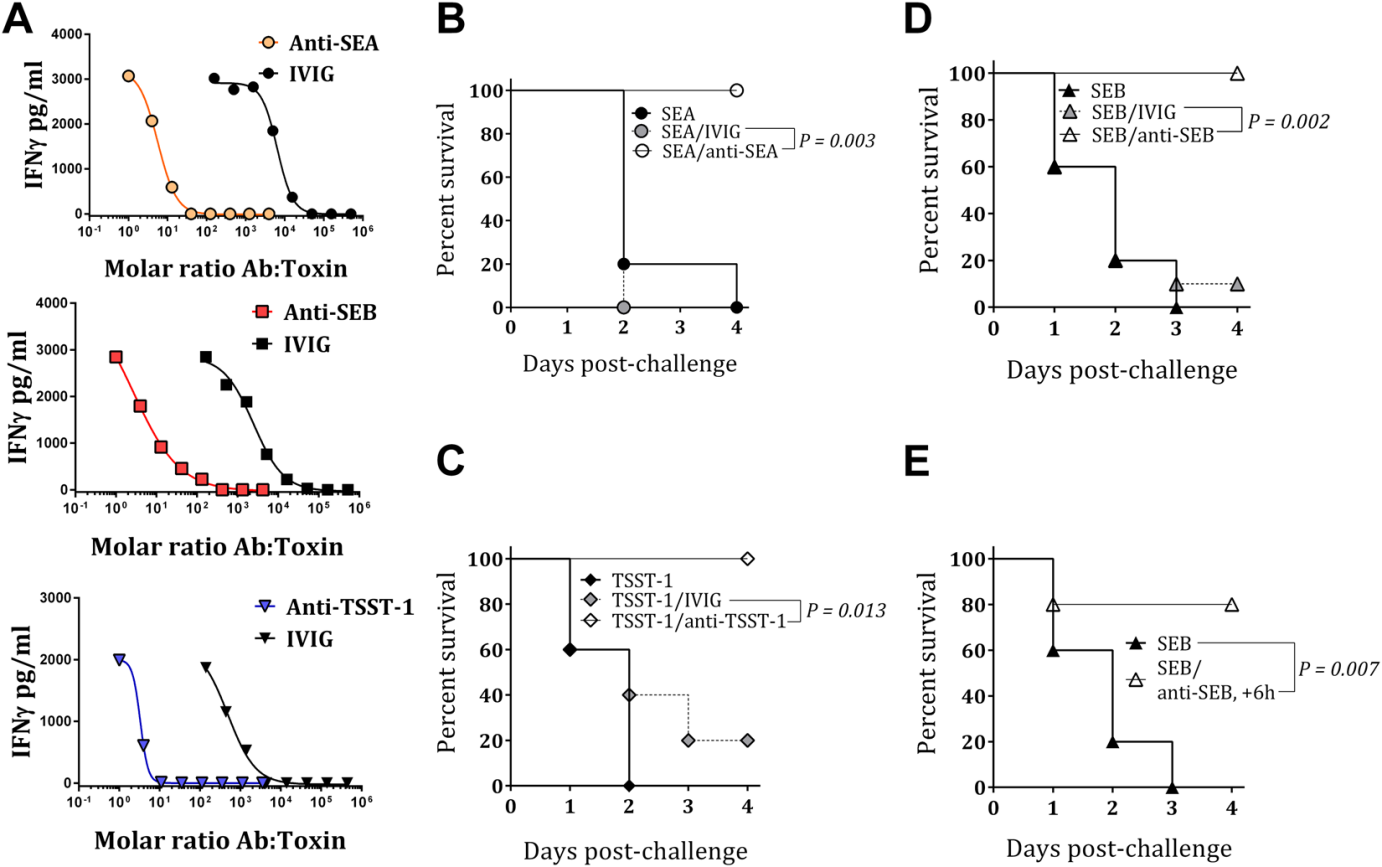
*In-vitro* and *in-vivo* neutralizing activity of purified human polyclonal antibodies. (**A**) Increased toxin neutralizing activity of purified human polyclonal antibodies compared to IVIG. (**B**–**D**) Active protection: mice were challenged with toxins either pre-incubated with purified human polyclonal Abs (open symbols) or IVIG (grey symbols) or with toxin only (black symbols). (**E**) Passive protection: mice were treated with anti-SEB pAbs 6 h post SEB challenge.

To further evaluate the potency of these natural anti-SAg antibodies we enriched polyclonal antibodies (pAbs) against SEA, SEB, and TSST-1 in IVIG (pooled from 28 lots) by affinity purification. The neutralizing activity of the preparations toward the respective toxins was then tested in PBMCs using IFNγ release as indication of superantigenicity. As shown in Fig. 2A, purified anti-SEA, anti-TSST-1, and anti-SEB pAbs exhibited approximately 1200-fold, 200-fold, and 900-fold enrichment of neutralizing activity compared to IVIG, respectively. With 50% inhibitory concentration (IC_50_) values in the range of 15–20 ng/ml (~0.5–0.6 nM) the specific antibodies appear to have very strong neutralization capacities. The purified antibodies were then tested for protection in toxin challenge animal models.

### Human polyclonal antibodies provide protection *in vivo* against toxin challenge

We tested the protective efficacy of purified human anti-SAg antibodies over a period of 5 days *in vivo* in the LPS potentiation model of toxic shock as previously described^42^. Approximately 1.25 µg/mouse of SEA, TSST-1 or SEB was either mixed with 125 µg of their homologous purified antibody or with 125 µg of IVIG (molar ratio: ~20 Ab: toxin) and incubated for 1 hour at RT before IP administration into Balb/c mice. After 4 h mice were challenged with 40 µg LPS. As shown in Fig. 2B–D, mice challenged with the combination of each toxin and its homologous affinity purified pAb (SEA/anti-SEA, SEB/anti-SEB or TSST/anti TSST-1) were 100% protected. In contrast, pre-incubation of IVIG and the toxin resulted in 0% survival for SEA (Fig. 2B), 20% survival for TSST-1 (Fig. 2C), and 10% survival for SEB (Fig. 2D). None of the control mice that received toxin alone survived the challenge.

To test a potential therapeutic effect of polyclonal antibodies, mice were challenged first with 10 LD_50_ of SEB followed by 40 µg LPS, and then treated with 125 µg of purified human anti-SEB pAbs 6 hours post SEB challenge. All control mice, receiving no antibody, died within 3 days, whereas 80% of the mice receiving anti-SEB pAbs survived (Fig. 2E). These data suggest that (i) human anti-SAg antibodies can protect against SAg mediated TSS post toxin exposure, and (ii) the concentration of anti-SAg antibodies in IVIG is probably insufficient to provide meaningful protection in this TSS model.

### Broad neutralization towards homologous and heterologous SAgs with antibodies to SEA, SEB and TSST-1

**Figure 3:**
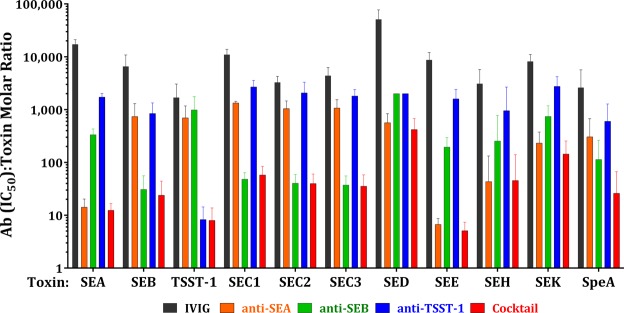
Broad neutralization of various superantigens. Anti-SEA, - SEB and -TSST-1 pAbs purified from IVIG, IVIG and a cocktail of purified pAbs were tested for neutralizing activity of a wide range of staphylococcal superantigens and streptococcal SpeA.

A major challenge for targeting SAgs by vaccines and immunotherapeutics is the fact that various *S*. *aureus* strains can produce more than 20 different SAgs. Despite low sequence identity in the primary structure, SAgs have highly conserved three-dimensional structure^43^ and cross-reactivity between anti-SAg antibodies has been reported^44^. Since SEA, SEB, and TSST-1 represent a divergent set of SAgs, we investigated if a combination of anti-SEA, -SEB and -TSST-1 pAbs could neutralize a broad spectrum of SAgs. We evaluated the neutralizing activity of individual affinity purified human anti-SEA, -SEB and -TSST-1 pAbs, or a cocktail of the three antibodies (from here on referred to as cocktail) towards a wide range of staphylococcal SAgs as well as the related streptococcal SpeA in toxin neutralization assays using PBMCs from five healthy donors. IFNγ production in PBMC culture was used as a readout and the IC_50_ values were determined for each stimulating toxin. Figure 3 shows the molar ratio at IC_50_ over toxin concentration as a measure of the relative homologous and heterologous neutralizing potency of each of the purified pAbs, the cocktail, and IVIG (mean ratio and SD of five donors). As expected, anti-SEA, anti-SEB, and anti-TSST-1 pAbs displayed the highest activity (lowest Ab:toxin ratio) against the homologous SAg with the respective IC_50_: toxin ratio being about 10–30. All affinity purified antibodies exhibited a higher potency toward heterologous toxins as compared with IVIG (black bars in Fig. 3 showing the highest ratios). Anti-SEA pAbs (orange bars, Fig. 3) exhibited strong cross neutralization of SEE and SEH with ratios of 7, and 30 respectively. Anti-SEB (green bars) cross neutralizing activity was evident towards SEC. 1–3, and to a lesser extent against SEE, SEH, and SpeA. TSST-1 (blue bars) displayed the lowest level of cross neutralization. However, the cocktail (red bars) was cross-neutralizing towards all superantigens at levels equivalent or better than individual antibodies. The lowest level of neutralization by the cocktail was observed against SED and SEK with mean ratios of ~300 and 130 respectively (IC_50_ values: 4.4 and 0.72 μg/ml respectively). However, these levels still represented ~127 and 57-fold increased neutralizing potency, respectively, as compared to IVIG.

In summary, these data indicate that broad neutralization of multiple SAgs can be achieved with a combination of potent neutralizing antibodies to SEA, SEB, and TSST-1 suggesting that such responses may be elicited with a multivalent toxoid vaccine against these three toxins.

### Generation and characterization of a fusion toxoid vaccine for SEA, SEB, and TSST-1

SAgs cross-link the MHC class II on the surface of antigen presenting cells (APC) with the TCR on the surface of T cells. By engineering three point mutations in the MHC binding surface of SEA, SEB, and TSST-1 inactivated toxoids: SEA_L48R/D70R/Y92A_, SEB_L45R/Y89A/Y94A_ (STEBVax), and TSST-1 -TSST_L30R/D27A/I46A_) were generated and tested in animal models of toxic shock^45–51^. We sought to examine if these three toxoids can be fused into a single polypeptide as a vaccine candidate.

**Figure 4:**
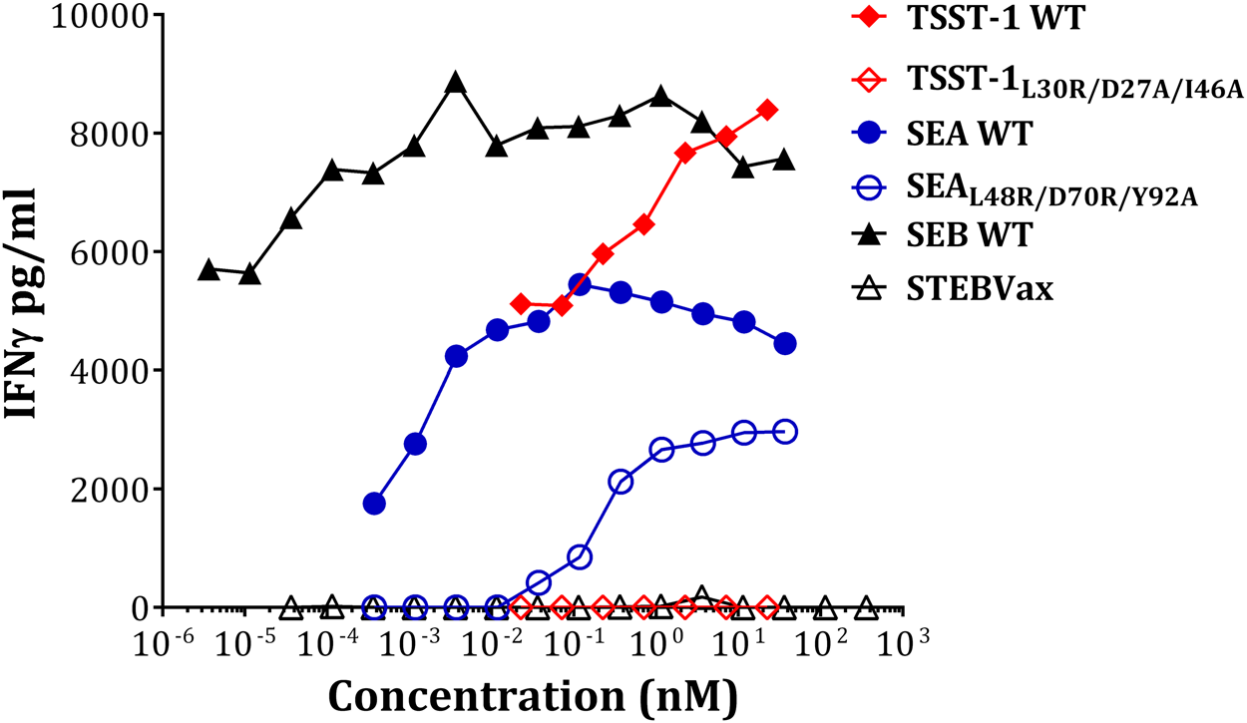
PBMC stimulation (interferon-gamma response) profile of wild-type super antigens TSST-1, SEA, SEB and their respective mutants TSST-1_L30R/D27A/I46A_, SEA_L48R/D70R/Y92A_ and STEBVax.

Superantigens are known for their potentially life-threatening toxicity, and hence a superantigen vaccine must be carefully analyzed for its safety. While the safety of STEBVax has been extensively evaluated including a phase I clinical trial that we recently conducted^52^, the safety of SEA and TSST-1 toxoids has not been extensively studied. We were also concerned that fusion of the three toxoids may exacerbate some residual superantigenic activity. Thus, we first evaluated the IFNγ response of PBMC from healthy human donors to wildtype SEA, SEB and TSST-1 in comparison to their mutant counterparts. As shown in Fig. 4, TSST-1_L30R/D27A/I46A_ as well as STEBVax showed no IFNγ response at the highest concentrations tested, however, SEA_L48R/D70R/Y92A_, although largely attenuated, exhibited clear IFNγ response at concentrations above 0.1 nM indicating that it retains some residual activity. Previously it was reported that mutation of H225 in the high-affinity MHC binding site of SEA reduces the ability of the toxin to activate T cells^53,54^. Therefore, we introduced H225 A as an additional safety mutation into the SEA-triple mutant to generate SEA_L48R/D70R/Y92A/H225A_.

**Figure 5:**
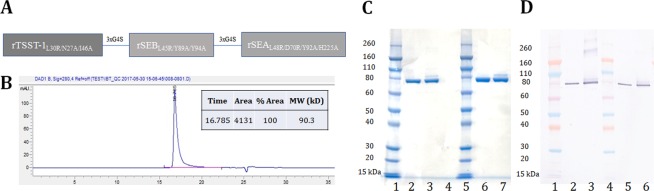
Biophysical characterization of TBA_225_. (**A**) Sequence of TBA_225_ (**B**) SEC-HPLC of TBA_225_ (**C**) SDS-PAGE for TBA in comparison to TBA_225_. Lanes 1 and 5: Protein ladder. Lane 2 and Lane 3: 1 µg of reducing and non-reducing versions of TBA. Lane 6 and Lane 7: 1 µg of reducing and non-reducing versions of TBA_225_ respectively. (**D**) Western blot for TBA in comparison to TBA_225_ at reducing as well as non-reducing buffer conditions. Lanes 1 and 4: Protein ladder. Lanes 2 and 3: 25 ng of reducing and non-reducing versions of TBA. Lanes 5 and 6: 25 ng of reducing and non-reducing versions of TBA_225_.

Two fusion cDNAs were constructed fusing the coding regions for the three mutants for TSST-1, SEB, and SEA, with and without the H225A mutation, named TBA and TBA_225_, respectively. The individual proteins were spaced with a flexible linker consisting of three GGGGS repeats (3xG4S) (Fig. 5A**)**. The fusion proteins were expressed in *E*. *coli* and purified by column chromatography. The fusion proteins showed a single peak in SEC-HPLC with an apparent molecular weight of 90.3 kDa (Fig. 5B) and a single band in SDS-PAGE (Fig. 5C) and Western blot (Fig. 5D) analysis. Thermostability of the constructs was evaluated by differential scanning fluorimetry (DSF). TSST-1_L30R/D27A/I46A_, STEBVax and SEA_L48R/D70R/Y92A_ had melting temperatures of 63.8, 53.5 and 38 °C respectively. We found that TBA and TBA_225_ have an average melting temperature of 41.8 °C and 39.2 °C, respectively indicating that the additionally introduced mutation only had marginal impact on the stability of SEAVax and consequently TBA_225_ (Table 1).

**Table 1:** Melting temperature of toxoids determined by DSF.

Protein	Avg Tm (°C) ± Std. Error
TBA	41.8 ± 0.58
TBA_225_	39.2 ± 0.29
TSST_L30R/D27A/I46A_	63.8 ± 0.29
STEBVax	53.5 ± 0.0
SEA_L48R/D70R/Y92A_	38.0 ± 0.0
TSST-1	64.5 ± 0.0
SEB	60.7 ± 0.0
SEA	62.0 ± 0.0

**Figure 6:**
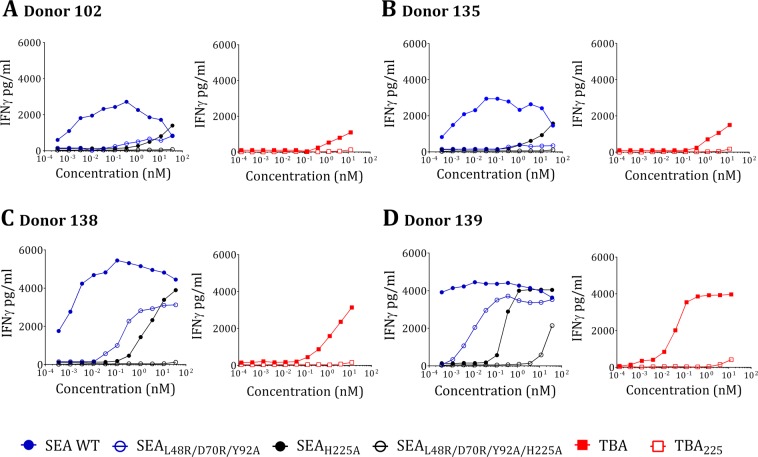
PBMC stimulation (interferon-gamma response) profile to donors (**A**)102, (**B**)135, (**C**)138, (**D**)139 by SEA and its mutants SEA_L48R/D70R/Y92A_, SEA_H225A_, SEA_L48R/D70R/Y92A/H225A_ as well as TBA and TBA_225_.

Safety of TBA and TBA_225_ along with individual toxoids was evaluated in PBMCs from four donors. As shown in Fig. 6A–D, neither SEA_L48R/D70R/Y92A_ or SEA_H225A_ were fully attenuated. In contrast, SEA_L48R/D70R/Y92A/H225A_ was completely inactive except in a highly sensitive donor (Fig. 6D) that displayed a low response at high concentration of the toxoid. TBA displayed various levels of residual toxicity in all four donors, while TBA_225_ was fully attenuated, even more than SEA_L48R/D70R/Y92A/H225A_ (Fig. 6D). The data indicate that TBA_225_ mutant represents a safe vaccine candidate.

### Immunogenicity of the superantigen fusion toxoids

**Figure 7:**
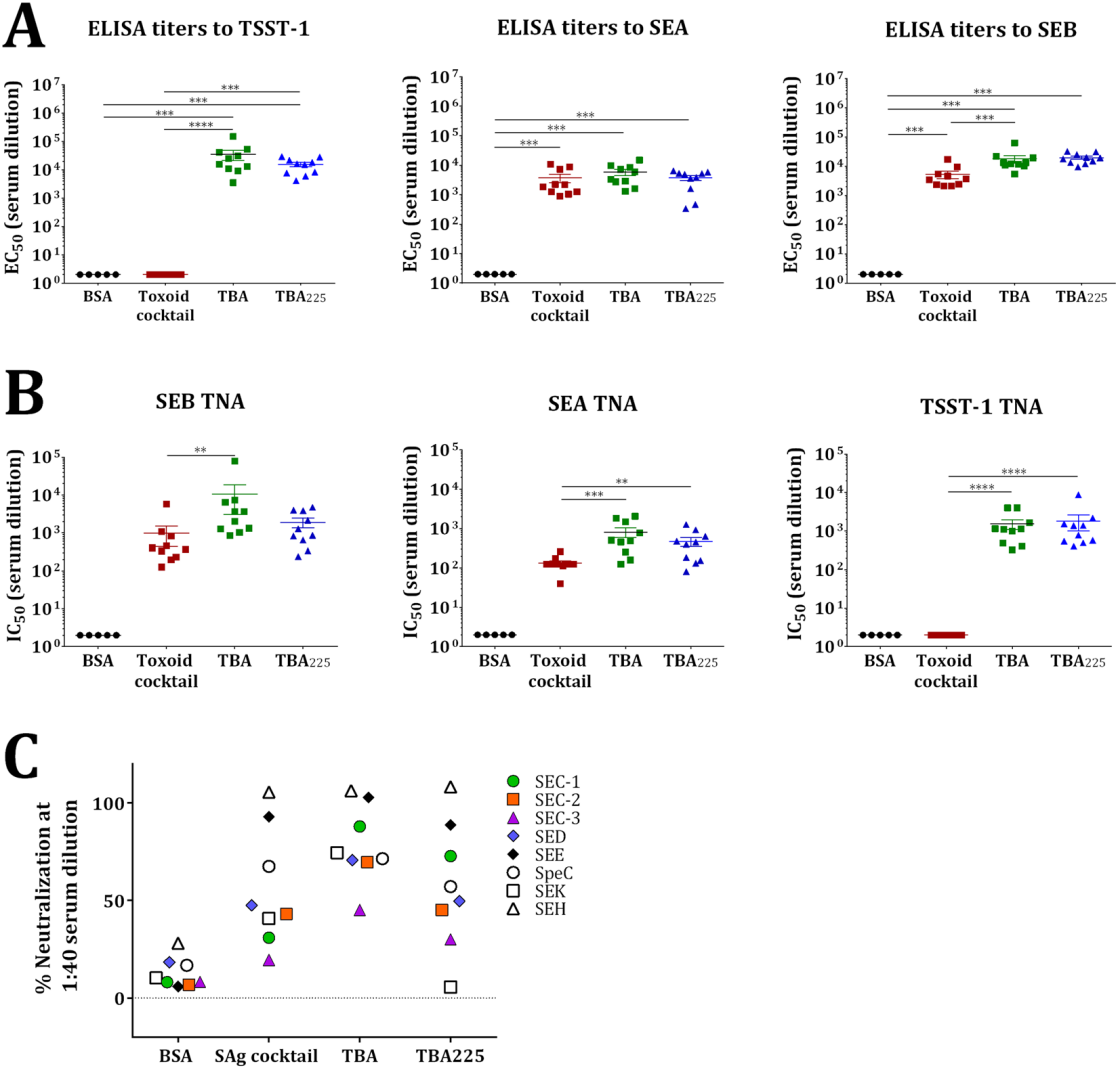
ELISA and TNA titers of mouse sera. Mice were immunized with BSA, toxoid cocktail, TBA or TBA_225_ and serum samples were tested for (**A**) Binding towards SEB, SEA and TSST-1 by ELISA, expressed as EC_50_ values, (**B**) Neutralization of SEB, SEA and TSST-1 determined by inhibition of IFN-γ release by human PBMCs, expressed as IC_50_ values and (**C**) Cross-neutralization of other superantigens expressed as percentage neutralization values at 1:40 serum dilution.

We next evaluated the immunogenicity of TBA and TBA_225_ in comparison with a cocktail of the three toxoids using Alhydrogel as adjuvant. Groups of 10 Balb/c mice were immunized three times on days 0, 14, and 28 with the toxoid cocktail (6.6μg each), TBA (20 μg), or TBA_225_ (20 μg) or BSA (20μg) as control. Sera were collected on day 35 and used to determine the ELISA binding and toxin neutralization activity (TNA) titers. As shown in Fig. 7A, all three formulations induced high levels of IgG titers against SEA and SEB. Notably, no anti-TSST-1 IgG titer was observed in the group immunized with the toxoid cocktail while both fusion proteins induced high titers. This was likely due to TSST-1’s poor adsorption to Alhydrogel under the conditions used for formulation (Supplementary Fig. S1), however, an immunological interference in the mixture cannot be ruled out. Similarly, high levels of TNA titers were observed against SEA and SEB with the cocktail and against all three antigens with the fusion proteins. TBA and TBA_225_ elicited comparable binding and neutralizing titers to both SEA and SEB in comparison to SAg toxoid cocktail and TBA. Consistent with the ELISA data the cocktail failed to elicit neutralizing titers to TSST-1, while TBA and TBA_225_ elicited similar neutralizing titers to TSST-1 (Fig. 7B), indicating that a fusion-protein can compensate for the low immunogenicity of individual TSST-1 toxoid. In addition, TBA_225_ elicited cross-reactive antibodies to SEC-1, SEC-2, SpeC and SEK as well as neutralizing titers to SEC-1, SEC-2, SpeC, SEH and SEE determined at 1:100 and 1:40 serum dilution, respectively (Fig. 7C).

### TBA_225_ immunization protects against toxin challenge

**Figure 8:**
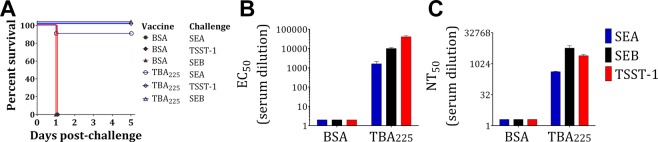
Immunogenicity and efficacy of TBA225. Balb/c mice were immunized with BSA or TBA225, subjected to toxin (SEA, SEB or TSST-1) challenge and monitored for (**A**) survival. Pooled serum samples were tested for (**B**) binding and (**C**) neutralizing titers towards SEA, SEB and TSST-1.

We next sought to evaluate the ability of TBA_225_ to provide protection against toxin challenge in the TSS mouse model. Six groups of 10 Balb/c mice were immunized with either BSA as negative control or TBA_225_ as described above. Mice were then challenged with SEA, SEB, or TSST-1 on day 35 followed by LPS. While all BSA immunized mice succumbed to toxic shock within 24 h, TBA_225_ immunization provided 100% protection against SEB and TSST-1 and 90% protection against SEA (Fig. 8A). Analysis of day 35 sera showed that TBA_225_ immunized mice had high binding and neutralizing titers to SEA, SEB and TSST-1 in contrast to BSA immunized mice (Fig. 8B,C).

### TBA_225_ vaccine elicits cross-reactive and cross-neutralizing antibodies

**Figure 9:**
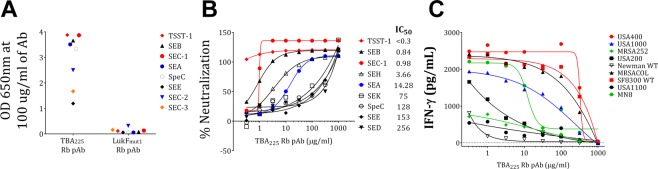
(**A**) Binding, (**B**) Neutralization by TBA_225_ polyclonal antibody to superantigens SEA, SEB, TSST-1, SEC-1, SEC-2, SEC-3, SEH, SEK, SEJ, and SEI, (**C**) Reduction in interferon-gamma production by bacterial supernatants from strains: USA400, USA1000, MRSA252, USA200, Newman WT, MRSACOL, SF8300 WT, USA 1100, and MN8 upon exposure to TBA_225_ rabbit polyclonal.

To evaluate the breadth of reactivity of antibodies elicited by TBA_225_ we generated polyclonal antisera in rabbits and purified total IgG by protein A chromatography. Polyclonal antibodies raised against another staphylococcal toxin, an attenuated Leukocidin F (LukF) molecule (LukF_mut1_)^36^ was used as control. The rabbit polyclonal anti-TBA_225_ IgG showed binding to all SAgs tested (Fig. 9A). Anti-TBA_225_ IgG was also tested in TNA assays against purified TSST-1, SEA, SEB, SEC-1, SEH, SEK, SEE, SED, and the streptococcal SpeC and was shown to neutralize all these toxins with different potencies (Fig. 9B). Highest level of cross-neutralization was evident for SEC-1, and SEH, while IC_50_ values for SEK, SEE, SED, and SEE were above 100 μg/ml (Fig. 9B). As expected LukF_mut1_ polyclonal did not elicit any binding (Fig. 9A) or neutralizing titers (data not shown).

We next examined if the anti-TBA_225_ pAbs can inhibit the stimulation of human PBMCs by culture supernatants of various *S*. *aureus* clinical strains. PBMCs were stimulated in presence of increasing concentration of anti-TBA_225_ or anti-LukF_mut1_ pAbs with overnight culture supernatants of USA300 (SF8300) (1:4000), USA400 (MW2) (1:16), USA200 (1:12), USA1100 (1:400), MRSA252 (1:4), MRSA COL (1:4000), Newman (1:4000), and MN8 (1:40). After a 48 h incubation IFNγ concentration in the supernatants was measured by quantitative ELISA. As expected different strains induced various levels of IFNγ production. USA400, MN8, COL, and SF8300 exhibited the highest level of IFNγ induction (Fig. 9C), while USA1000 and USA200 showed intermediate levels and MRSA252, USA100, and Newman induced low levels of IFNγ. Anti-TBA_225_ IgG was able to inhibit the stimulatory activity in all these supernatants to various degrees (Fig. 9C) indicating that SAg mediated activities in these supernatants are being neutralized.

## Discussion

The objective of the presented work was to evaluate if broad neutralization of most prominent staphylococcal SAgs is possible with a single subunit vaccine. We studied the breadth of neutralizing and cross-neutralizing antibodies elicited by natural exposure to staphylococcal superantigens. Our findings show that human antibodies to SEA, SEB, and TSST-1 can neutralize multiple related SAgs and can provide broader neutralization when combined. Concentration of these antibodies in human plasma appears to be fairly low and their elevation to therapeutically meaningful levels would require immunization. To this end we generated a fusion peptide toxoid vaccine (TBA_225_) consisting of toxoids for SEA, SEB, and TSST-1 and demonstrated its efficacy against toxin challenge *in vivo* and its ability to elicit broad cross-neutralizing antibodies against a wide range of SAgs. Our findings are important for development of a future multivalent toxoid vaccine for *S*. *aureus* infection and prevention or treatment of superantigen induced diseases and SA infections in general.

Presence of antibodies to SAgs in normal human plasma and IVIG preparations has been previously reported^38–40,55^. However, to our knowledge the anti-SAg antibody content in healthy human plasma has not been quantitatively determined. In a cohort of 30 healthy plasma donors we found that all individuals had detectable levels of antibodies to SEA, SEB, TSST-1, SED, as well as SpeA and SpeC with circulating concentrations ranging from 100 ng/ml to low μg/ml (Fig. 1A). Based on the dose response curve of the affinity purified antibodies (Fig. 2A), full neutralization of SEA, SEB, and TSST-1 requires 50, 300, and 20-fold molar excess of the respective IVIG-derived polyclonal antibodies. Thus, for example, a concentration of 0.694 μg/ml (4.6 nM) anti-SEB in blood (median of 30 samples, Fig. 1A) can fully detoxify ~0.015 nM of circulating SEB (~0.5 ng/ml). Similarly, the plasma sample in our cohort with the highest anti-SEB titer (4.5 μg/ml, Fig. 1A) and the plasma with the lowest concentration (0.1 μg/ml) would be able to detoxify a maximum blood concentration of ~3.1 and 0.07 ng/ml SEB, respectively. Little is known about the local and systemic levels of SAgs produced during infection. Azuma *et al*. reported circulating SAg concentration in ICU patients in the range of 0.01–0.15 ng/ml^56^. Clearly at least a portion of the population is unable to fully detoxify these levels of circulating toxins. In addition, given that SAgs are produced at the site of infection and then released into the circulation, it is conceivable that the local concentration of SAgs are exponentially higher and far above the neutralizing capacity of circulating natural antibodies as we determined here.

Human immunoglobulin preparations have been used for treatment of streptococcal and staphylococcal TSS with limited success and their utility remains controversial^30–33^. We determined the concentration of anti-SEB antibodies in 28 lots of IVIG and found the median to be 121 μg/g IVIG (range 82–188). Based on this analysis, a dose of 400 mg/kg IVIG would deliver 2.3–5.3 mg of anti-SEB antibodies to a 70 Kg patient yielding added concentrations of 0.5–1 μg/ml specific antibody. Thus, the commonly used doses of IVIG therapy only incrementally increase the circulating concentrations of anti-SAg antibodies.

Our data indicate that vaccination is required to achieve a safe excess of SAg neutralizing antibodies in humans. Anamnestic response to vaccines often leads to exponential increase in antibody titers^57–59^. We recently completed a Phase I clinical trial of a monovalent SAg vaccine and a component of TBA_225_, STEBVax (SEB_L45R/Y89A/Y94A_)^55^. In this study, a single dose of STEBVax (2.5 μg or greater) led to an average of ~40 fold elevation of anti-SEB titer (range 3.7–194 fold) within 2 weeks^55^. In addition, Schwameis *et al*. tested a recombinant TSST-1 toxoid in healthy human volunteers and demonstrated a strong antibody response to the vaccine^60^. These data indicate that the baseline titers against SAgs can be exponentially elevated by vaccination as opposed to incremental increase achieved by IVIG treatment.

A combination of human polyclonal antibodies against SEA, SEB, and TSST-1 neutralized a wide range of SAgs at levels better or equivalent to the individual antibodies (Fig. 3). Based on this finding we generated a fusion protein consisting of toxoids for these three SAgs, named TBA_225_, and demonstrated its full attenuation with respect to superantigenic toxicity (Fig. 6). The fusion vaccine elicited homologous and heterologous antibody responses at levels generally higher than a cocktail of the three toxoids, indicating that the fusion not only has simplified the vaccine but also increased its immunogenicity (Fig. 7). TBA_225_ provided full protection against the three superantigens in a toxic shock model (Fig. 8A) and vaccine-elicited antibodies neutralized a wide range of SAgs including SEK, the primary SAg produced by the currently circulating USA300 lineage. TBA_225_-elicited antibodies also neutralized the intoxication of human PBMCs by crude culture supernatants of multiple clinically relevant *S*. *aureus* isolates including USA300 (Fig. 9).

To date efforts towards development of vaccines for *S*. *aureus* have been focused on surface antigens, modeled on the success of capsular polysaccharide vaccines for several bacterial agents. However, all these efforts have failed to achieve the clinical endpoint of preventing staphylococcal infections. At least one of these vaccines, V710, had even a deleterious impact on the outcome of *S*. *aureus* infection in vaccinated individuals including statistically significant higher multiorgan failure in the vaccine arm (31 vs 17 events; *P* = 0.04) and the observation that the V710 recipients who suffered a *S*. *aureus* infection were 5 times more likely to die than patients that received the placebo vaccine^6^. A post-hoc study performed on a subset of available sera from these patients suggested that low pre-vaccination IL-2 and IL-17A levels might have predisposed these patients to catastrophic mortality upon *S*. *aureus* infection^7^. Furthermore, two studies in mice^61^ and rabbits^62^ also indicate that immunization with surface antigens can induce a detrimental immune response. Collectively, these data indicate that a dysregulated immune response may be the primary cause of lack of response to vaccines or vaccine mediated enhancement of *S*. *aureus* disease.

Toxoids represent a promising alternative as “anti-virulence” vaccines. This is consistent with reports that lower level of antibodies against toxins correlate with severity of disease^29,63^. Previously, we have reported strong protection against multiple syndromes of *S*. *aureus* using attenuated vaccines for pore forming toxins alpha hemolysin and leucocidin components^36,64–66^. Beside their prominent role in TSS, SAgs also impact the virulence of SA through induction of a local excessive inflammatory response, immune subversion by inducing apoptosis of T and B cells^11,12^ and modulation of the function of regulatory T cells (Tregs)^13–15^, innate lymphoid cells (ILCs)^16^, and unconventional T cells such as γδ T cells^17,18^, NKT cells^19–21^, and mucosa associated invariant T (MAIT) cells^22^. SAgs are also implicated in the pathogenesis of neonatal exanthematous disease, infective endocarditis, sepsis, and atopic dermatitis^23^. Several groups have reported partial protection against *S*. *aureus* infections in various animal models using vaccines or antibodies against SEB^67^, SEA^68^, TSST-1^69^, SEC.^70^ and SEK^71^. SAgs are implicated in sepsis based on higher prevalence in septicemia-causing isolates^72^, significantly higher and more frequent detection of SAgs in patients with sepsis^56^, and serological studies in bacteremic patients^29^ and are known to alter the human immune system. Thus, the superantigen vaccine presented in this report can be a valuable addition to attenuated pore forming toxoids to generate a multivalent toxin-based vaccine for *S*. *aureus*.

## Supplementary information

Supplementary Figure

## References

[1] Fowler VG, Proctor RA (2014). Where does a Staphylococcus aureus vaccine stand?. Clin Microbiol Infect.

[2] Oliveira, D., Borges, A. & Simoes, M. Staphylococcus aureus Toxins and Their Molecular Activity in Infectious Diseases. *Toxins (Basel)* **10** (2018). 10.3390/toxins10060252 PMC6024779 29921792

[3] Bassetti M, Nicco E, Mikulska M (2009). Why is community-associated MRSA spreading across the world and how will it change clinical practice?. Int J Antimicrob Agents.

[4] Bradley SF (2005). Staphylococcus aureus pneumonia: emergence of MRSA in the community. Semin Respir Crit Care Med.

[5] Pier GB (2013). Will there ever be a universal Staphylococcus aureus vaccine?. Hum Vaccin Immunother.

[6] Fowler VG (2013). Effect of an investigational vaccine for preventing Staphylococcus aureus infections after cardiothoracic surgery: a randomized trial. JAMA.

[7] McNeely TB (2014). Mortality among recipients of the Merck V710 Staphylococcus aureus vaccine after postoperative S. aureus infections: an analysis of possible contributing host factors. Hum Vaccin Immunother.

[8] Proctor RA (2015). Recent developments for Staphylococcus aureus vaccines: clinical and basic science challenges. Eur Cell Mater.

[9] Schlievert PM (1993). Role of Superantigens in Human Disease. The Journal of Infectious Diseases.

[10] Bohach GA, Fast DJ, Nelson RD, Schlievert PM (1990). Staphylococcal and streptococcal pyrogenic toxins involved in toxic shock syndrome and related illnesses. Crit Rev Microbiol.

[11] Mahlknecht U, Herter M, Hoffmann MK, Niethammer D, Dannecker GE (1996). The toxic shock syndrome toxin-1 induces anergy in human T cells *in vivo*. Hum Immunol.

[12] Hofer MF (1996). Differential effects of staphylococcal toxic shock syndrome toxin-1 on B cell apoptosis. Proc Natl Acad Sci USA.

[13] Lee J (2018). Induction of Immunosuppressive CD8(+)CD25(+)FOXP3(+) Regulatory T Cells by Suboptimal Stimulation with Staphylococcal Enterotoxin C1. J Immunol.

[14] Tanriver Y, Martin-Fontecha A, Ratnasothy K, Lombardi G, Lechler R (2009). Superantigen-activated regulatory T cells inhibit the migration of innate immune cells and the differentiation of naive T cells. J Immunol.

[15] Tilahun AY, Chowdhary VR, David CS, Rajagopalan G (2014). Systemic inflammatory response elicited by superantigen destabilizes T regulatory cells, rendering them ineffective during toxic shock syndrome. J Immunol.

[16] Broker, B. M., Mrochen, D. & Peton, V. The T Cell Response to Staphylococcus aureus. *Pathogens* **5** (2016). 10.3390/pathogens5010031 PMC4810152 26999219

[17] Maeurer, M., Zitvogel, L., Elder, E., Storkus, W. J. & Lotze, M. T. Human intestinal V delta 1+ T cells obtained from patients with colon cancer respond exclusively to SEB but not to SEA. *Nat Immun* **14**, 188–197 (1995). 8696008

[18] Morita CT (2001). Superantigen recognition by gammadelta T cells: SEA recognition site for human Vgamma2 T cell receptors. Immunity.

[19] Hayworth JL (2012). CD1d-independent activation of mouse and human iNKT cells by bacterial superantigens. Immunol Cell Biol.

[20] Rieder SA, Nagarkatti P, Nagarkatti M (2011). CD1d-independent activation of invariant natural killer T cells by staphylococcal enterotoxin B through major histocompatibility complex class II/T cell receptor interaction results in acute lung injury. Infect Immun.

[21] Szabo PA (2017). Invariant Natural Killer T Cells Are Pathogenic in the HLA-DR4-Transgenic Humanized Mouse Model of Toxic Shock Syndrome and Can Be Targeted to Reduce Morbidity. J Infect Dis.

[22] Shaler, C. R. *et al*. MAIT cells launch a rapid, robust and distinct hyperinflammatory response to bacterial superantigens and quickly acquire an anergic phenotype that impedes their cognate antimicrobial function: Defining a novel mechanism of superantigen-induced immunopathology and immunosuppression. *PLoS Biol* **15**, e2001930 (2017). 10.1371/journal.pbio.2001930 PMC5478099 28632753

[23] Salgado-Pabon, W. *et al*. Superantigens are critical for Staphylococcus aureus Infective endocarditis, sepsis, and acute kidney injury. *MBio* **4** (2013). 10.1128/mBio.00494-13 PMC3747586 23963178

[24] Spaulding AR (2013). Staphylococcal and Streptococcal Superantigen Exotoxins. Clinical Microbiology Reviews.

[25] Spaulding AR (2012). Immunity to Staphylococcus aureus secreted proteins protects rabbits from serious illnesses. Vaccine.

[26] Holtfreter S (2004). egc-Encoded superantigens from Staphylococcus aureus are neutralized by human sera much less efficiently than are classical staphylococcal enterotoxins or toxic shock syndrome toxin. Infect Immun.

[27] Papageorgiou AC, Acharya KR (2000). Microbial superantigens: from structure to function. Trends Microbiol.

[28] Bavari S, Ulrich RG, LeClaire RD (1999). Cross-reactive antibodies prevent the lethal effects of Staphylococcus aureus superantigens. J Infect Dis.

[29] Adhikari RP (2012). Lower Antibody Levels to Staphylococcus aureus Exotoxins Are Associated With Sepsis in Hospitalized Adults With Invasive S. aureus Infections. J Infect Dis.

[30] Donovan S, Bearman GM (2014). Use of intravenous immunoglobulin in critically ill patients. Curr Infect Dis Rep.

[31] Kadri SS (2017). Impact of Intravenous Immunoglobulin on Survival in Necrotizing Fasciitis With Vasopressor-Dependent Shock: A Propensity Score-Matched Analysis From 130 US Hospitals. Clin Infect Dis.

[32] Schlievert PM (2001). Use of intravenous immunoglobulin in the treatment of staphylococcal and streptococcal toxic shock syndromes and related illnesses. J Allergy Clin Immunol.

[33] Shah PJ, Vakil N, Kabakov A (2015). Role of intravenous immune globulin in streptococcal toxic shock syndrome and Clostridium difficile infection. Am J Health Syst Pharm.

[34] Niesen FH, Berglund H, Vedadi M (2007). The use of differential scanning fluorimetry to detect ligand interactions that promote protein stability. Nat Protoc.

[35] Berthold F (1981). Isolation of human monocytes by Ficoll density gradient centrifugation. Blut.

[36] Karauzum H (2013). Structurally Designed Attenuated Subunit Vaccines for S. aureus LukS-PV and LukF-PV Confer Protection in a Mouse Bacteremia Model. PLoS One.

[37] Chen AE (2009). Discordance between Staphylococcus aureus nasal colonization and skin infections in children. Pediatr Infect Dis J.

[38] Takei S, Arora YK, Walker SM (1993). Intravenous immunoglobulin contains specific antibodies inhibitory to activation of T cells by staphylococcal toxin superantigens [see comment]. J Clin Invest.

[39] Toungouz M, Denys CH, De Groote D, Dupont E (1995). *In vitro* inhibition of tumour necrosis factor-alpha and interleukin-6 production by intravenous immunoglobulins. Br J Haematol.

[40] Yanagisawa C, Hanaki H, Natae T, Sunakawa K (2007). Neutralization of staphylococcal exotoxins *in vitro* by human-origin intravenous immunoglobulin. J Infect Chemother.

[41] Parsonnet J (2005). Prevalence of toxic shock syndrome toxin 1-producing Staphylococcus aureus and the presence of antibodies to this superantigen in menstruating women. J Clin Microbiol.

[42] Stiles BG, Bavari S, Krakauer T, Ulrich RG (1993). Toxicity of staphylococcal enterotoxins potentiated by lipopolysaccharide: major histocompatibility complex class II molecule dependency and cytokine release. Infect Immun.

[43] Schlievert PM (1995). Molecular structure of staphylococcus and streptococcus superantigens. J Clin Immunol.

[44] Bavari S, Ulrich RG, LeClaire RD (1999). Cross-Reactive Antibodies Prevent the Lethal Effects of Staphylococcus auveus Superantigens. The Journal of Infectious Diseases.

[45] Ulrich RG, Olson MA, Bavari S (1998). Development of engineered vaccines effective against structurally related bacterial superantigens. Vaccine.

[46] Ulrich, R. G., Olson, M. A. & Bavari, S. Bacterial superantigen vaccines (2004).

[47] Ulrich, R. G., Olson, M. A. & Bavari, S. Bacterial superantigen vaccines (2002).

[48] Ulrich, R. G. Fusion protein of streptococcal pyrogenic exotoxins (2006).

[49] Ulrich, R. G. Altered superantigen toxins (2010).

[50] Aman, M. J., Adhikari, R. P., Shulenin, S., Holtsberg, F. W. & Karauzum, H. Toxoid Peptides Derived from Phenol Soluble Modulin, Delta Toxin, Superantigens, and Fusions Thereof (2016).

[51] Ulrich, R. G., Olson, M. A. & Bavari, S. Bacterial superantigen vaccines (2011).

[52] Chen, W. H. *et al*. The safety and immunogenicity of a parenterally administered structure-based rationally modified recombinant Staphylococcal enterotoxin B protein vaccine, STEBVax. *Clinical and Vaccine Immunology* (2016). 10.1128/CVI.00399-16 PMC5139602 27707765

[53] Hudson KR (1995). Staphylococcal enterotoxin A has two cooperative binding sites on major histocompatibility complex class II. The Journal of Experimental Medicine.

[54] Kozono, H., Parker, D., White, J., Marrack, P. & Kappler, J. Multiple binding sites for bacterial superantigens on soluble class II MHC molecules. *Immunity* **3**, 187–196 (1995). 10.1016/1074-7613(95)90088-8 7648392

[55] Chen WH (2016). Safety and Immunogenicity of a Parenterally Administered, Structure-Based Rationally Modified Recombinant Staphylococcal Enterotoxin B Protein Vaccine, STEBVax. Clin Vaccine Immunol.

[56] Azuma K (2004). Detection of circulating superantigens in an intensive care unit population. Int J Infect Dis.

[57] Kim DS (2016). Immunogenicity and Safety of a Booster Dose of a Live Attenuated Japanese Encephalitis Chimeric Vaccine Given 1 Year After Primary Immunization in Healthy Children in the Republic of Korea. Pediatr Infect Dis J.

[58] Poovorawan Y (2013). Long-term anti-HBs antibody persistence following infant vaccination against hepatitis B and evaluation of anamnestic response: a 20-year follow-up study in Thailand. Hum Vaccin Immunother.

[59] Rennels M (2004). Dosage escalation, safety and immunogenicity study of four dosages of a tetravalent meninogococcal polysaccharide diphtheria toxoid conjugate vaccine in infants. Pediatr Infect Dis J.

[60] Schwameis M (2016). Safety, tolerability, and immunogenicity of a recombinant toxic shock syndrome toxin (rTSST)-1 variant vaccine: a randomised, double-blind, adjuvant-controlled, dose escalation first-in-man trial. Lancet Infect Dis.

[61] Karauzum H (2017). Lethal CD4 T Cell Responses Induced by Vaccination Against Staphylococcus aureus Bacteremia. J Infect Dis.

[62] Spaulding, A. R. *et al*. Vaccination Against Staphylococcus aureus Pneumonia. *J Infect Dis* (2014). 10.1093/infdis/jit823 PMC4038136 24357631

[63] Fritz, S. A. *et al*. A Serologic Correlate of Protective Immunity Against Community-Onset Staphylococcus aureus Infection. *Clin Infect Dis* (2013). 10.1093/cid/cit123 PMC3641868 23446627

[64] Adhikari RP (2012). Novel Structurally Designed Vaccine for S. aureus alpha-Hemolysin: Protection against Bacteremia and Pneumonia. PLoS One.

[65] Adhikari RP (2015). Antibodies to S. aureus LukS-PV Attenuated Subunit Vaccine Neutralize a Broad Spectrum of Canonical and Non-Canonical Bicomponent Leukotoxin Pairs. PLoS One.

[66] Adhikari RP, Thompson CD, Aman MJ, Lee JC (2016). Protective efficacy of a novel alpha hemolysin subunit vaccine (AT62) against Staphylococcus aureus skin and soft tissue infections. Vaccine.

[67] Varshney, A. K. *et al*. Staphylococcal Enterotoxin B-Specific Monoclonal Antibody 20B1 Successfully Treats Diverse Staphylococcus aureus Infections. *J Infect Dis* (2013). 10.1093/infdis/jit421 PMC3836467 23922375

[68] Nilsson IM, Lee JC, Bremell T, Ryden C, Tarkowski A (1997). The role of staphylococcal polysaccharide microcapsule expression in septicemia and septic arthritis. Infect Immun.

[69] Hu DL (2003). Vaccination with nontoxic mutant toxic shock syndrome toxin 1 protects against Staphylococcus aureus infection. J Infect Dis.

[70] Mattis DM (2013). Engineering a soluble high-affinity receptor domain that neutralizes staphylococcal enterotoxin C in rabbit models of disease. Protein Eng Des Sel.

[71] Aguilar, J. L. *et al*. Monoclonal antibodies protect from Staphylococcal Enterotoxin K (SEK) induced toxic shock and sepsis by USA300 Staphylococcus aureus. *Virulence*, **0** (2016). 10.1080/21505594.2016.1231295 PMC5626247 27715466

[72] Humphreys H (1989). Enterotoxin production by Staphylococcus aureus isolates from cases of septicaemia and from healthy carriers. J Med Microbiol.

