# Supplementary material for: TBA225, a fusion toxoid vaccine for protection and broad neutralization of staphylococcal superantigens
Source: Sci Rep. 2019 Mar 1;9:3279. doi: 10.1038/s41598-019-39890-z (PMC6397225; doi:10.1038/s41598-019-39890-z)

**Supplementary Information to:**

**TBA_225_, a fusion toxoid vaccine for protection and broad neutralization of staphylococcal superantigens**

Arundhathi Venkatasubramaniam^1^, Rajan P. Adhikari^1^, Thomas Kort^1^, Grant C. Liao^1^, Shawn Conley^1^, Laura Abaandou^1^, Yoshikuni Onodera^2^, Shweta Kailasan^1^, Subramaniam Krishnan^1^, Didier M. Djagbare^1^, Frederick W. Holtsberg^1^, Hatice Karauzum^1^, M. Javad Aman^1#^

^1^Integrated Biotherapeutics, Inc., Rockville, MD, USA; ^2^Daiichi Sankyo, Tokyo, Japan.

# Corresponding author: [jaman@integratedbiotherapeutics.com](mailto:jaman@integratedbiotherapeutics.com)

**Supplemental figure 1**

SDS run for supernatants from various antigen: adjuvant incubation as follows – 10µg of each antigen along with varying ratios of adjuvant Alhydrogel: A) SAg cocktail with 1:5, 1:10, 1:15, 1:20, and 1:25 of adjuvant, B) TBA with 1:5, 1:6, 1:7, 1:8 and 1:9 of adjuvant C) TBA_225_ with 1:5, 1:10, 1:15, 1:20, 1:25 of adjuvant D) SEA/ STEBVax with 1:5, 1:10 of Alhydrogel and E) TSST-1 with 1:5, 1:10, 1:15, 1:20, 1:25 of Alhydrogel.


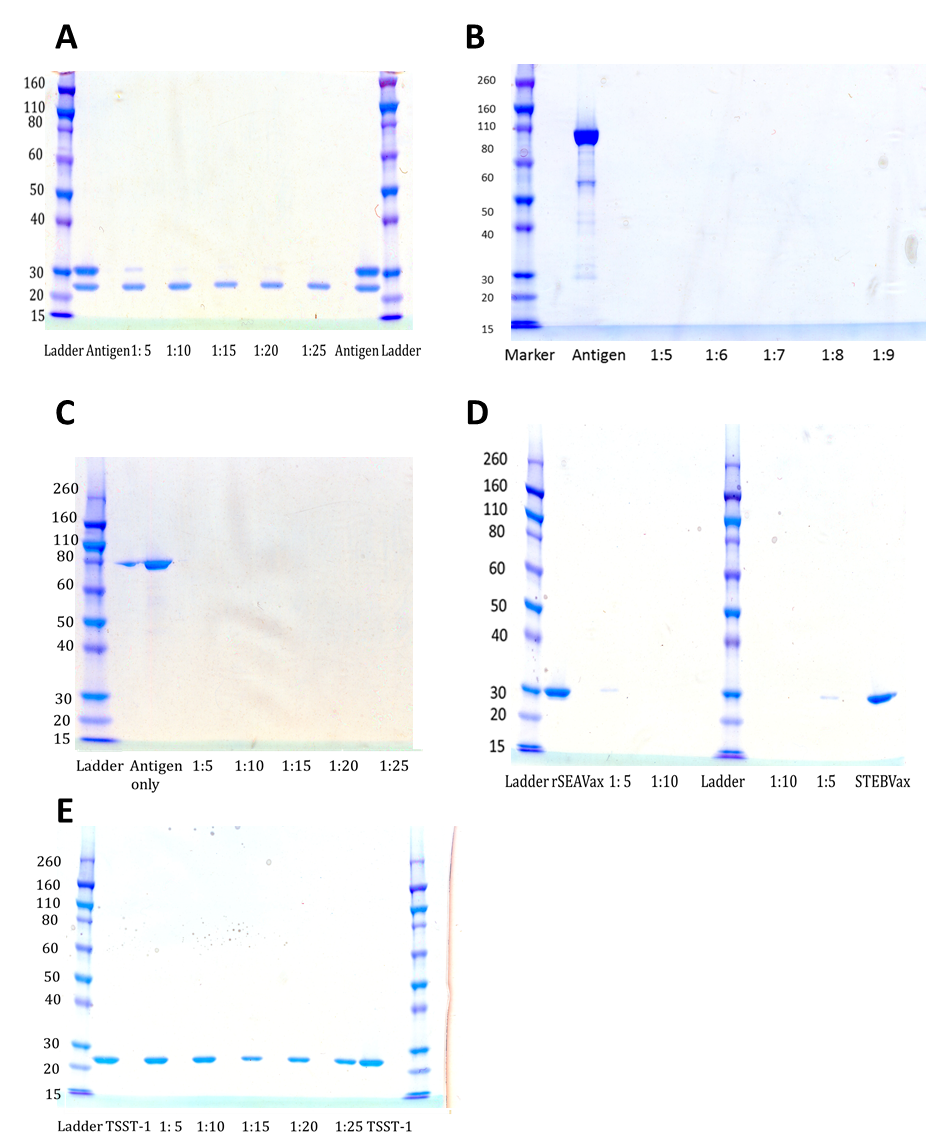

Supplement: Supplementary file 1 — Supplementary Figure [file 41598_2019_39890_MOESM1_ESM.docx]
